# Perampanel attenuates oxidative stress and pyroptosis following subarachnoid hemorrhage via the SIRT3/FOXO3α pathway

**DOI:** 10.1038/s41598-023-48802-1

**Published:** 2023-12-03

**Authors:** Hongqiao Yang, Changgeng Ding, Ming Cheng, Zhengwei Sheng, Lei Chen, Junhui Chen, Yuhai Wang

**Affiliations:** 1 Wuxi Clinical College of Anhui Medical University, Wuxi, China; 2 The Fifth Clinical College of Anhui Medical University, Hefei, China

**Keywords:** Immunology, Neuroscience

## Abstract

Subarachnoid hemorrhage (SAH) occurs most commonly after rupture of an aneurysm, resulting in high disability and mortality due to the absence of effective therapy. Its subsequent stage, early brain injury (EBI), promotes the sustainable development of injury in the brain and ultimately leads to poor prognosis. As a new antiepileptic drug, the effect of perampanel on EBI after SAH is unknown. Pyroptosis, a process of inflammatory programmed cell death, has been confirmed in most studies to play a substantial role in aggravating SAH-post EBI. Similarly, oxidative stress is closely involved in neuronal pyroptosis and the pathophysiological mechanism of SAH-post EBI, leading to a devastating outcome for SAH patients. Nonetheless, no studies have been conducted to determine whether perampanel reduces pyroptosis and oxidative stress in the context of SAH-induced EBI. Rat SAH model via endovascular perforation was constructed in this study, to assess the neuroprotective effect of perampanel on SAH-post EBI, and to clarify the possible molecular mechanism. By means of the neurological score, brain edema detection, FJB staining, immunofluorescence, WB, ELISA, and ROS assay, we found that perampanel can improve neuroscores and reduce brain edema and neuronal degeneration at 24 h after SAH; we also found that perampanel reduced oxidative stress, neuronal pyroptosis, and inhibition of the SIRT3-FOXO3α pathway at 24 h after SAH. When 3-TYP, an inhibitor of SIRT3, was administered, the effects of perampanel on the SIRT3-FOXO3a pathway, antioxidant stress, and neuronal pyroptosis were reversed. Taken together, our data indicate that perampanel attenuates oxidative stress and pyroptosis following subarachnoid hemorrhage via the SIRT3/FOXO3α pathway. This study highlights the application value of perampanel in subarachnoid hemorrhage and lays a foundation for clinical research and later transformation of perampanel in SAH.

## Introduction

As a subtype of stroke, subarachnoid hemorrhage (SAH) primarily induced by aneurysm rupture, is characterized by high morbidity and mortality rates and remaining neurological deficits^1^. Although the treatment has been greatly improved in past decades, the unsatisfactory prognosis is still a difficult problem for doctors and researchers^2^. Brain injury that occurs within 72 h after SAH, also known as early brain injury (EBI), is closely related to the prognosis of SAH^3^. The mechanism for SAH-post EBI is particularly complex and involves oxidative stress, inflammation, mitochondrial dysfunction, pyroptosis, autophagy, etc.^4^. Pyroptosis, a particular inflammatory programmed cell death, has been confirmed in most studies to play an essential role in aggravating SAH-post EBI^5^. Similarly, oxidative stress is closely associated with neuronal pyroptosis and plays a vital role in the pathophysiological mechanism of SAH-post EBI, leading to a devastating outcome for SAH patients^6^. It has been reported that reactive oxygen species (ROS) production after SAH is a key step in NLRP3-mediated pyroptosis^7^. Although the “chief culprit” aneurysm can be processed perfectly by interventional or clipping treatment, the secondary injury as a result of aneurysm ruptures still progresses^8^. Currently, many drugs targeting these mechanisms have been researched and developed, but there is no significant improvement in the outcome of SAH patients^1,8^. In this situation, other viable strategies for improving neurological function in SAH patients must be implemented and initiated.

Perampanel has been used as an oral anti-epileptic drug in many counties and regions, providing highly selective and non-competitive antagonism to the alpha-amino-3-hydroxy-5-methyl-4-isoxazolepropionic acid (AMAP) receptor^9^. In recent years, perampanel has been proven to play a neuroprotective role in hemorrhagic ischemic stroke models by regulating the destruction of the blood–brain barrier (BBB)^10,11^. Perampanel can also attenuate oxidative stress and inflammation after traumatic brain injury (TBI)^9^, but the specific mechanism is not yet clear. Meanwhile, evidence suggests that perampanel alleviates neurovascular unit disruption following TBI in a sirtuin3 (SIRT3)-dependent manner^12^. It has been demonstrated that perampanel alleviates damage to the blood–brain barrier in the SAH model by antagonizing the activation of AMPAR and then plays a protective role in early brain injury^10^. Interestingly, the prognosis of SAH is increasingly reported to be associated with some types of epileptic discharges^13^. In the clinic, anti-epileptic drugs are often used to prevent secondary damage to the brain as a result of seizures after SAH. Therefore, exploring more mechanisms of perampanel in reducing brain injury after subarachnoid hemorrhage will further reflect the application value of perampanel. Notably, it is not clear whether perampanel attenuates brain injury after subarachnoid hemorrhage in other ways, such as antioxidative stress and the pyroptosis pathway.

SIRT3, a type of deacetylase, is located in mitochondria and exerts its powerful function dependent on NAD^+^^14^. SIRT3 can regulate cellular energy metabolism and stress response to help damaged cells endure “difficult times” due to its powerful biological functions including maintaining mitochondrial homeostasis, resisting oxidative stress and regulating autophagy^14,15^. Recent studies have shown that SIRT3 is strongly related to NLRP3-mediated pyroptosis^16,17^. However, it remains to be determined whether and how SIRT3 can regulate neuronal pyroptosis to alleviate early brain injury after SAH. The transcription factor, Forkhead box protein O3 (FOXO3α), is a central regulatory molecule for cellular homeostasis, stress response and lifespan, as it regulates various stress responses to nutrient deficiency, hypoxia, oxidative stress, heat shock and DNA damage^18^. FOXO3α in the cytoplasm, as one of the targeted molecules of SIRT3, is deacetylated into the activated state. Activated FOXO3α can enter the nucleus from the cytoplasm to regulate the expression of manganese superoxide dismutase (MnSOD), catalase (CAT), and other genes, affecting mitochondrial respiration and ROS clearance^18,19^. However, we do not know whether the SIRT3-FOXO3α pathway can modulate the degree of oxidative stress in the brain after SAH. Based on this evidence, we hypothesized that perampanel could attenuate oxidative stress and pyroptosis following subarachnoid hemorrhage via the SIRT3/FOXO3α pathway. Therefore, we conduct a series of experiments to verify this hypothesis.

## Materials and methods

### SAH models

This study was performed under the National Institutes of Health's guidelines for the care and use of laboratory animals. The Ethics Committee of The 904th Hospital of PLA of Wuxi Clinical School of Anhui Medical University approved all experimental protocols. Adult male Sprague–Dawley rats, weighing 280–320 g, were obtained from Hangzhou Medical College. The rats were constructed for the SAH model through endovascular perforation and following the protocol in our previous study^20^. And 5% isoflurane (RWD, Guangdong, China) in the N_2_O/O_2_ mixture (1:1) was used to anesthetized rats, followed by 2.5% isoflurane with a N_2_O/O_2_ (1:1) mixture face mask to maintain anesthesia. The sham group was constructed using the same procedure, without endovascular puncture.

### Experimental Design and animal groups

#### Experimental design

**Figure 1:**
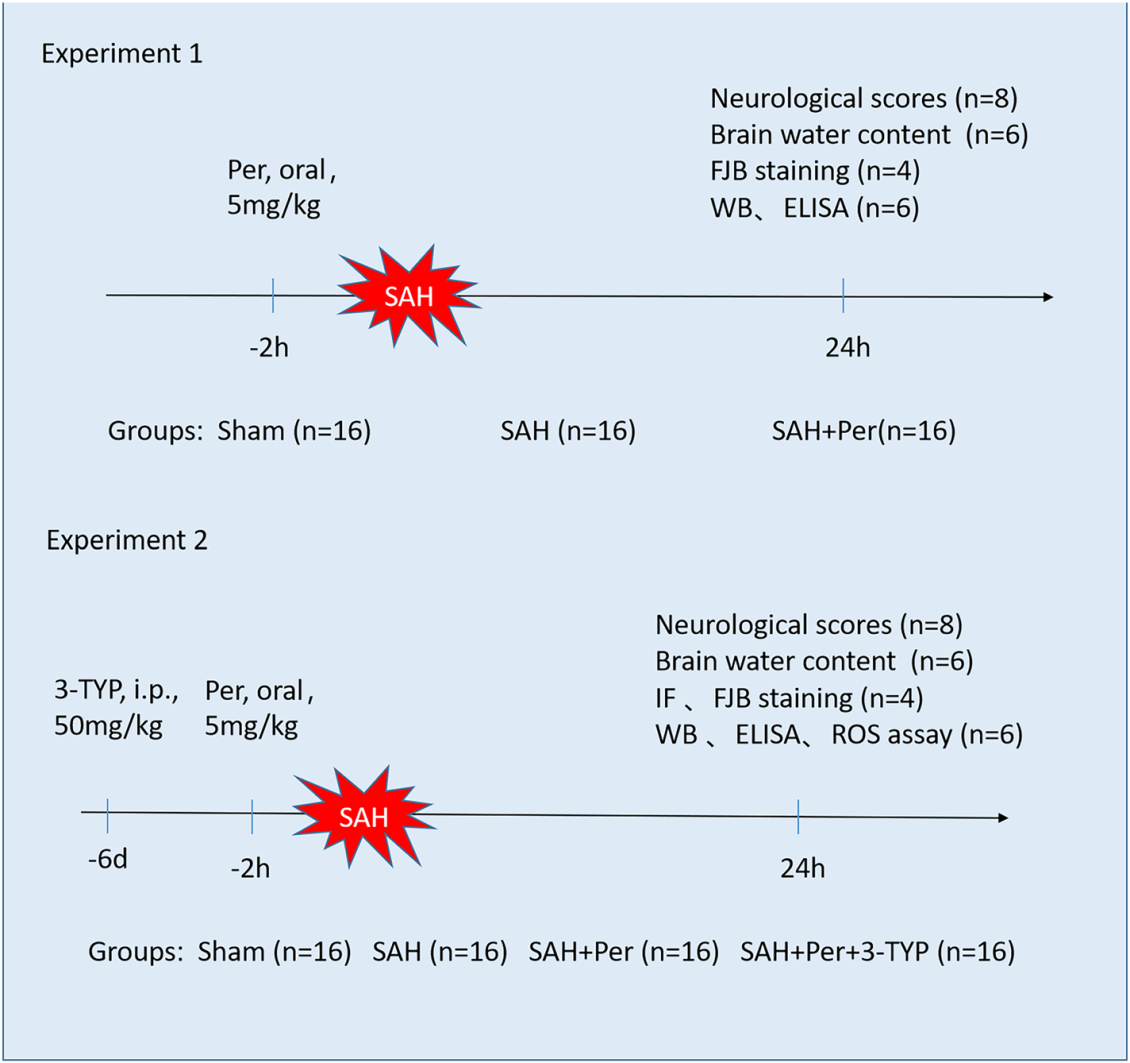
Experimental design.

**Experiment 1** To verify the neuroprotective effect of perampanel, 48 rats (68 rats were subjected to surgery, with 20 rats eliminated due to demise or SAH grade ≤ 7) were randomly divided into 3 groups: Sham group, SAH group and SAH + Per group, with 16 rats in each group (as shown in Fig. 1 experiment 1). 8 rats in each group were used to evaluate neurological scores (the modified Garcia score and the beam balance test), while 6 were used to measure the bran water content (BWC). In addition, 4 rats were in each group for detection of FJB staining, and 6 rats for Western blot (WB) assay of the alterations of SIRT3 and FOXO3α. Additionally, 6 rats (shared with WB assay) were used for ELISA to determine the changes in the inflammatory factors IL-1β and IL-18. The tests began 24 h after successful modelling.

**Experiment 2** To explore whether perampanel suppresses oxidative stress and NLRP3-induced pyroptosis through triggering SIRT3-mediated signaling pathway. 64 rats (86 rats were subjected to surgery, with 22 rats eliminated due to demise or SAH grade ≤ 7) were randomly divided into four groups, sham, SAH, SAH + Per (5 mg/kg), and SAH + Per + 3-TYP (50 mg/kg), with 16 rats in each group (as shown in Fig. 1 experiment 2). For the evaluation of neurological scoring, 8 rats were used; for brain water content, 6 rats were used; for FJB staining and immunofluorescence (IF) staining, 4 rats were used; for Western blotting, 6 were used. Additionally, 6 rats (shared with WB assay) were in each group for the measurement of ROS levels, and 6 rats (shared with WB assay) were used for ELISA. The tests began 24 h after successful modelling.

#### Animal groups

Rats in Sham group underwent the sham operation, with vehicle pre-treatment. Rats in SAH group underwent the SAH operation, with vehicle pre-treatment. Rats in SAH + Per group underwent the SAH operation, with perampanel/vehicle pre-treatment. Rats in SAH + Per + 3-TYP group were subjected to the SAH operation, with perampanel/3-TYP pre-treatment.

### Drug administration

Parampanel (Weicai, China) was taken orally at a 5 mg/kg dose 2 h before SAH ictus. Before modeling, equal volumes of 3-TYP (50 mg/kg) and vehicle (1% ethanol) were intraperitoneally (i.p.) given, at a dose every two days, for a total of three doses. The dosage regimens of perampanel and 3-TYP were based on previous studies^9,21^.

### Neurological score evaluation

Rat neurological function was assessed at 24 h after SAH, based on the modified Garcia scoring system and beam balance test, by two observers who were blinded to the study^22^. In brief, the modified Garcia test comprises six subtests. The three subtests of spontaneous movement, movement symmetry of limbs, and forelimb outstretching are scored from 0 to 3, while the other three tests, climbing ability, body proprioception, and response to vibrissae touch, were scored on a scale of 1–3, resulting in a total score ranging from 3 to 18. The beam balance test was conducted, to assess rats' walking ability in one minute on a 15-mm-wide wooden beam. The average score was calculated according to the walking ability of three consecutive scores from 0 to 4. The higher the score, the better the neurological function.

### SAH grade

The severity of SAH was defined by a separate observer who was blinded to the experiment, using SAH grade as previously reported^5^. In brief, the score ranges from 0 to 18 in this grading system based on the size of the blood clot. The basal of the rats was sectioned into 6. Each section was scored as follows: no subarachnoid hemorrhage recorded as 0, minimal subarachnoid hemorrhage recorded as 1, moderate blood volume with visible arteries recorded as 2, and clots blocking all arteries recorded as 3. Rats with scores below 8, indicating mild SAH, were excluded.

### Brain water content

After anesthetization and decapitation, rat brains were rapidly removed 24 h-post SAH, and the wet weight was immediately obtained. Then brains were dried for 24 h at 105℃ and the dry weight was obtained. The brain water content was determined as follows: [(wet weight − dry weight)/wet weight] × 100%.

### Hematoxylin–eosin (HE) staining

HE staining was performed to observe the brain tissue pathological morphology, following a previously described protocol^20^. Rats were subjected to trans-cardiac perfusion with 500 ml 4% tissue fixation solution (P1110, Solarbio, China) 24 h after SAH. After more than 24 h of fixation, the rat brain tissue was dehydrated and embedded in paraffin to make a continuous coronal section with a thickness of 4–6 µm. The dewaxed rat brain sections were stained with routine HE staining, observed under an optical microscope and photographed.

### Immunofluorescence (IF) staining

IF staining was performed as previously described^22^. The rat brain tissue was dehydrated and paraffin embedded to make paraffin sections. Paraffin-embedded brain tissue Sects. (4 ~ 6 μm thickness) were heated with citrate EDTA antigen repair solution (P0086, Beyotime, China), in a microwave oven for 30 min. The sections were then sealed at room temperature for 30 min with 5% goat serum (SL038, SolarBioLife Science, China) and incubated overnight at 4℃ with the following primary antibodies: anti-GSDMD antibody (1:50, ab15515, Abcam) and anti-NeuN antibody (1:500, ab104224, Abcam). Subsequently, the sections were rinsed with phosphate-buffered saline (PBS), followed by incubation with the appropriate secondary antibodies. After washing with PBS, the nuclei were stained for 15 min with 4,6-diamidino-2-phenylindole (DAPI) (Sigma Aldrich, USA). Capturing of the images was carried on a fluorescence microscope (Leica, DMI8, Germany).

### FJB staining

The degree of neuronal injury in the ipsilateral (right) temporal cerebral cortex was evaluated by FJB staining which can identify degenerating neurons. According to a previous method^22^, the rat brain tissue was made into paraffin sections as described above, and the paraffin sections were treated with xylene, combined with gradient ethanol. The prepared FJB staining solution (Sigma‒Aldrich, United States) was used to incubate the sections at room temperature for 20 min. Then, the nuclei were dyed with DAPI dye for 10 min and rinsed with pure water. After air-drying, the slices were placed in xylene for 2 min, and then sealed with neutral resin. Finally, images of the ipsilateral temporal cortex were collected by fluorescence microscopy (Leica, DMI8, Germany). Statistics and analysis of FJB ( +) neurons were performed in a blinded manner.

### Measurement of ROS levels

The production of ROS was also measured by the ROS Assay kit (Nanjing Jiancheng, E004-1-1, China) according to the manufacturer’s instructions^7^. The ROS content in brain tissues was evaluated as fluorescence intensity/mg protein.

### ELISA

The levels of proinflammatory factors, including IL-1β and IL-18, were detected by an IL-1β assay kit and an IL-18 assay kit. The brain oxidative damage index level, including malondialdehyde (MDA), was analyzed using an MDA assay kit following the manufacturer’s instructions. The levels of antioxidant factors, including superoxide dismutase (SOD) and glutathione peroxidase activity (GSHPx), were measured by the commercial kits (Nanjing Jiancheng Bioengineering Institute, China), respectively, in accordance with the manufacturer’s instructions. Acetylated FOXO3α (Ac-FOXO3α) was measured by an Acetyl-FOXO3α assay kit (JCL005, China) for rats.

### Western blotting

Western blotting was conducted as previously described^20^. First, RIPA lysate (Beyotime, China) was used for protein extraction from the rat cerebral cortex, and then the protein concentration between groups was determined by the BCA method (Beyotime, China). Thirty micrograms of total protein were loaded into each group, and the same volume of 2X Laemmli sample buffer was added. Then, the samples were boiled (5 min) to denature the protein. The boiled protein was transferred from the gel to a PVDF membrane (Millipore, United States), after sodium dodecyl sulfate‒polyacrylamide gel electrophoresis. Then, the membrane was blocked and incubated with a properly diluted primary antibody in blocking buffer at 4 °C overnight. The membrane was washed with TBST three times (5 min each time), followed by an incubation with the recommended diluted HRP-conjugated secondary antibody for 1 h at room temperature. The membrane was then washed three times with TBST detergent for 5 min each time. Finally, the blots were visualized by an ECL kit (Beyotime, China) and quantified using ImageJ software (National Institutes of Health, USA). The primary antibodies included anti-SIRT3 (1:1000, Ab246522, Abcam); anti-FOXO3α (1:1000, AF6020, Affinity); anti-CAT (1:2000, 66765-1-Ig, Proteintech); anti-MnSOD (1:10,000, 66474-1-Ig, Proteintech); anti-NLRP3 (1:1000, DF7438, Affinity); anti-ASC (1:1000, DF6304, Affinity); anti-cleaved caspase 1 (1:1000, AF4022, Affinity); anti-N-GSDMD (1:1000, A22523, ABclonal); and anti-β-actin (1:1000, AF7018, Affinity).

### Statistical analysis

GraphPad Prism software (8.0, San Diego, CA, USA) was used for statistical analysis. All the data are expressed as the mean ± standard deviation (SD). After the normality test, the data between two groups were compared using Student's t test, whereas the differences among multiple groups were analyzed by one-way analysis of variance (ANOVA) followed by Tukey's post hoc test. A P value of less than 0.05 was deemed to indicate a significant difference.

### Statement

We declare that this study was performed in line with the ARRIVE (Animal Research: Reporting of In Vivo Experiments) guidelines.

## Results

### Mortality and SAH grade

**Figure 2:**
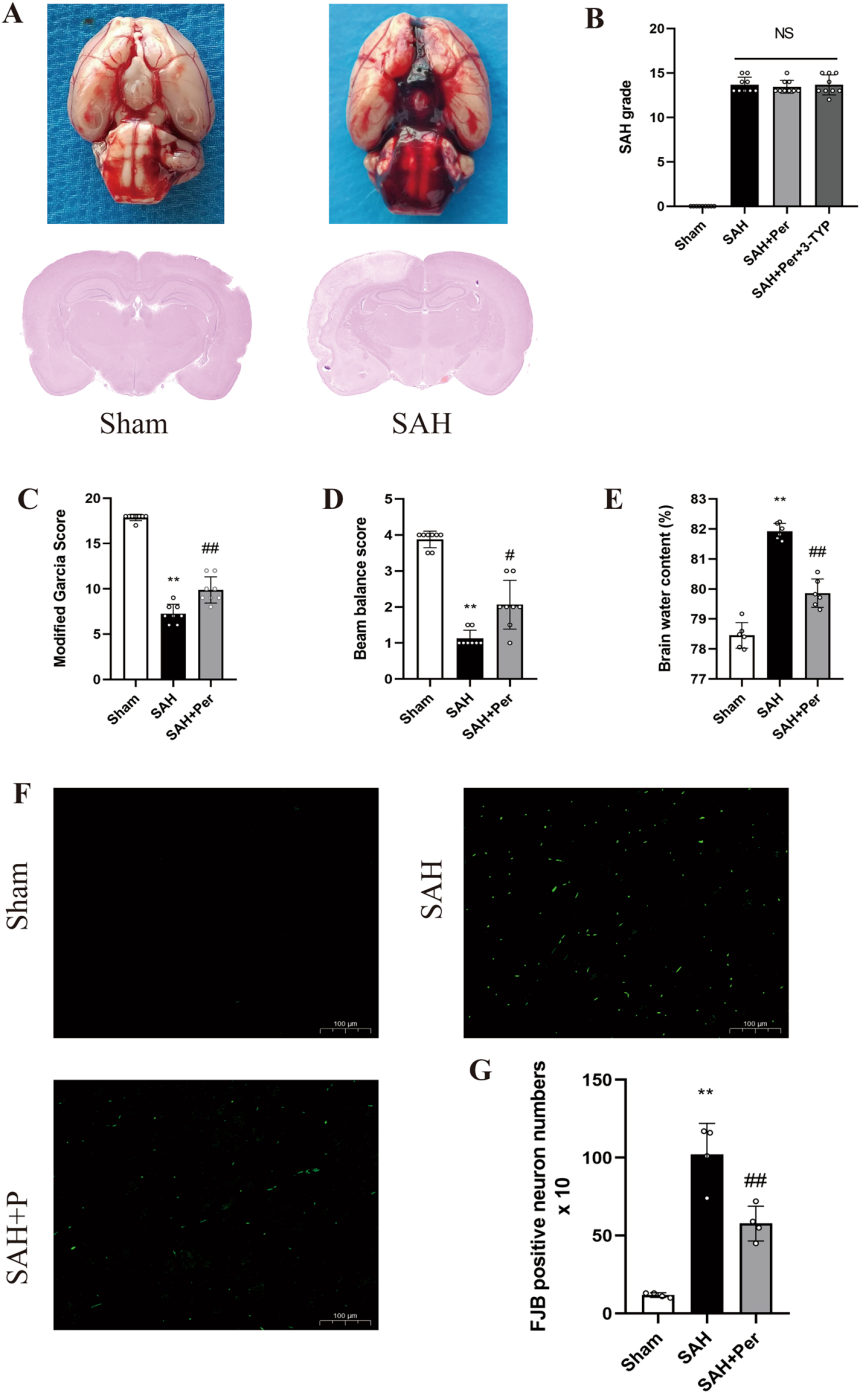
Typical images of the subarachnoid hemorrhage model and the therapeutic effect of oral perampanel on subarachnoid hemorrhage. (**A**) Representative photographs of the bottom of the rat brain and HE staining from sham and 24 h after SAH. (**B**) SAH grade scores 24 h after SAH in rats (n = 8). (**C-E**) Quantitative analysis of Modified Garcia Score (n = 8), Beam balance score (n = 8) and Brain water content (n = 6). (**F**) Representative images of FJB straining in the right temporal cerebral cortex of rats (magnification: 10X; scale bar: 100 µm). (**G**) Quantitative measurement of FJB staining (n = 4). Bars represent the mean ± SD; *P < 0.05, **P < 0.01 vs Sham group; ^#^P < 0.05, ^##^P < 0.01 vs. SAH group.

Representative images of the base of rat brains, as well as HE staining, from sham and 24 h-post SAH rats are shown in Fig. 2A. To guarantee rigorousness and consistency, 154 rats were recruited to participate in the experiment, of which 27 died within 24 h of SAH and 15 were eliminated due to the low SAH grade (less than 8). According to Table 1, no rats in the Sham group died, whereas 27 SAH rats were dead with a total mortality rate of 17.53% (27/154). The data show that perampanel did not decrease overall mortality. As shown in Fig. 2B, SAH grades did not differ significantly among the three SAH groups.

**Table 1:** The usage and mortality of animals in this study.

Groups	Mortality	Exclusion
Experiment 1		
Sham	0.00% (0/16)	0
SAH	24.00% (6/25)	3
SAH + Per	25.93% (7/27)	4
Experiment 2		
Sham	0.00% (0/16)	0
SAH	21.74 (5/23)	2
SAH + Per	20.83 (5/24)	3
SAH + Per + 3-TYP	17.39 (4/23)	3
Total		
Sham	0.00% (0/32)	0
SAH	17.53% (27/154)	15

### Perampanel ameliorates neurological deficits and neuronal degeneration after SAH

As shown in Fig. 2, the results of the modified Garcia scale and beam balance test were substantially reduced after SAH induction (Fig. 2C,D), indicating that SAH had a significant negative impact on the animals' neurological function and motor abilities. Surprisingly, perampanel treatment significantly reduced neurological deficits (modified Garcia score and beam balance score) after SAH. Meanwhile, FJB staining (Fig. 2F,G) demonstrated that the number of degenerated neurons increased substantially after SAH, whereas perampanel treatment significantly reduced the number of degenerated neurons.

### perampanel ameliorates the increase in brain water content and neuroinflammation at 24 h post-SAH

**Figure 3:**
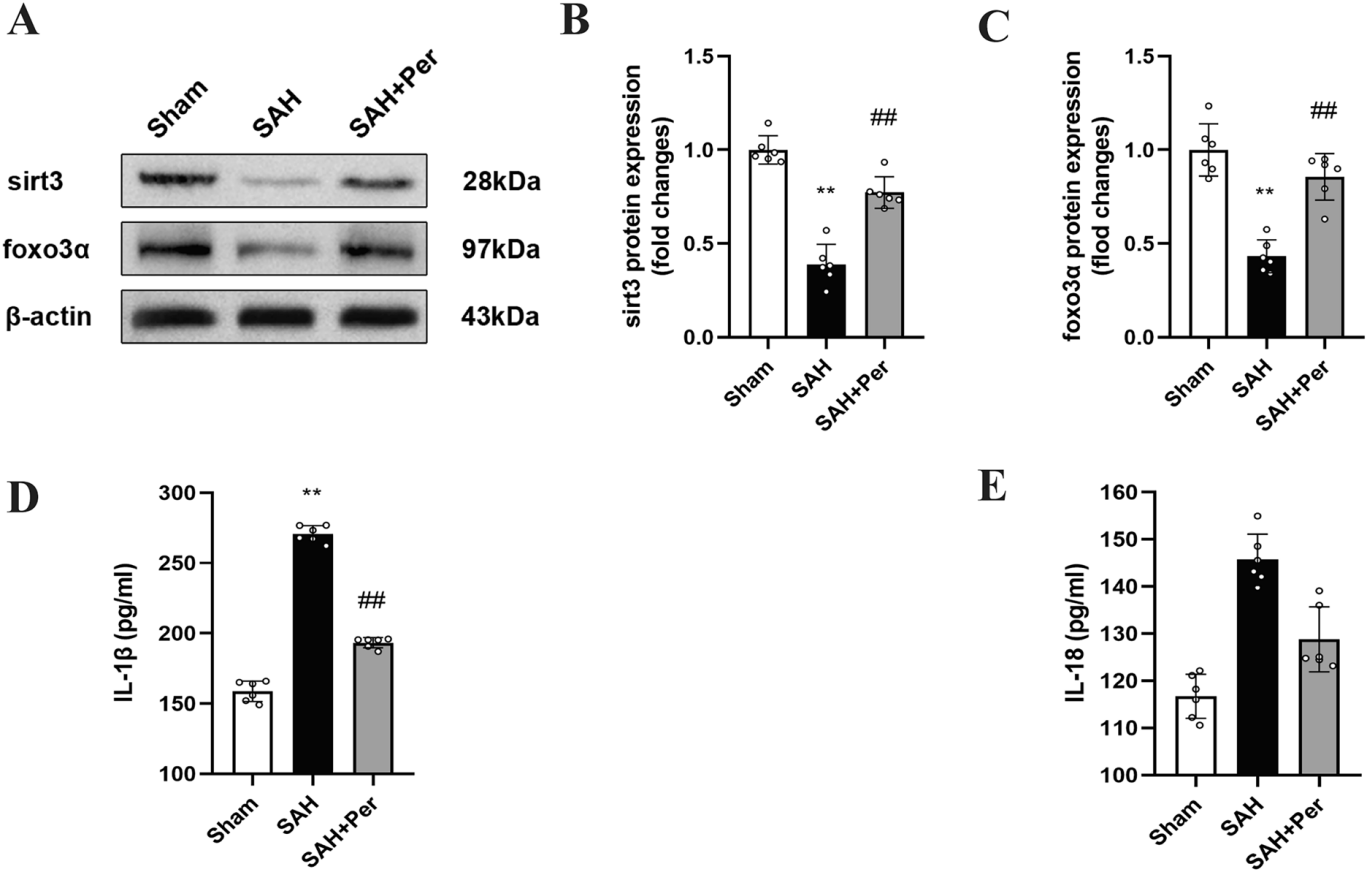
Perampanel alleviates the decrease in sirt3 and foxo3 and the increase in the inflammatory factors IL-1b and IL-18 at 24 h post-SAH. (**A**) Representative western blot images of SIRT3 and FOXO3a. (**B, C**) Quantitative analysis of the protein expression levels of SIRT3 and FOXO3a in each group (n = 6). (**D, E**) Quantitative analysis of the levels of the inflammatory factors IL-1β and IL-18 was detected by ELISA (n = 6). Bars represent the mean ± SD; *P < 0.05, **P < 0.01 vs Sham group; ^#^P < 0.05, ^##^P < 0.01 vs. SAH group.

The water content in the rat brain was determined for assessment of the brain edema severity due to the destruction of the BBB after SAH. The results (Fig. 2E) showed that rats developed significant brain edema at 24 h post-SAH. However, under perampanel treatment, the increase in brain water content was alleviated. These results show that perampanel reduces SAH damage to the blood–brain barrier. The ELISA results (Fig. 3D,E) showed that the inflammatory factors IL-1β and IL-18 increased 24 h after SAH and decreased after perampanel treatment, which indicates that neuroinflammation was reduced after perampanel treatment.

### 3-TYP reverses the effect of perampanel on the SIRT3-FOXO3α pathway at 24 h post-SAH

The WB results show the changes in the expression of SIRT3 and FOXO3α. Based on the WB results of Experiment 1 (shown in Fig. 3A–C), SIRT3 and FOXO3α expression in the right brain cortex of rats decreased in the SAH group, compared with those in the sham group. Conversely, SIRT3 and FOXO3α expression increased when the rats were given perampanel treatment. According to Experiment 2 (shown in Fig. 5A,B), the WB results showed that the expression of SIRT3 was reduced in the SAH + Per + 3-TYP group, compared with that in the SAH + Per group, which indicates that SIRT3 was effectively inhibited by 3-TYP treatment. The level of FOXO3α was increased in the SAH + Per group, compared with that in the SAH group. However, FOXO3α also decreased as SIRT3 decreased when 3-TYP was administered (Fig. 5C). As shown in Fig. 5D, when SAH occurred, the level of ac-FOXO3 increased; the level of ac-foxo3 declined when perampanel was administered; otherwise, the ac-FOXO3α level was increased in the SAH + Per + 3-TYP group compared with that in the SAH + Per group.

### Perampanel ameliorates oxidative stress at 24 h post-SAH

SAH caused obvious oxidative damage, as evidenced by the significant increases in ROS (Fig. 5G) and MDA (Fig. 5H), but decreases in GSH-Px and SOD activities (Fig. 5H–J) in the SAH group when compared with those in the sham group. The WB results (Fig. 5E,F) showed that the antioxidative enzymes Mn-SOD and CAT were both expressed at higher levels than in the sham group. After being treated with perampanel, the ROS assay shows that the fluorescence intensity indicating ROS content decreases when compared with the SAH group. Moreover, ELISA showed decreases in MDA and increases in GSH-Px and SOD activities in the SAH + Per group compared with those in the SAH group. The WB results showed that the antioxidative enzymes Mn-SOD and CAT were both expressed at lower levels in the SAH + Per group than those in the SAH group.

### Perampanel ameliorates NLRP3 inflammasome-induced neuronal pyroptosis at 24 h post-SAH

The occurrence of pyroptosis aggravates the inflammatory response in the brain and leads to an irreversible outcome after SAH. NLPR3 inflammatory activity plays an extremely important role in neuronal pyroptosis. The WB (Fig. 6A–E) and ELISA (Fig. 6G,H) results showed increases in the expression levels of NLRP3, ASC, cleaved caspase-1, N-GSDMD, IL-1β, and IL-18 compared with those in the sham group. There were decreases in the expression of NLRP3, ASC, cleaved caspase-1, N-GSDMD, IL-1β, and IL-18 in the SAH + Per group, compared with those in the SAH group. Moreover, double immunofluorescence staining (Fig. 6F) showed that there was an increase in the expression level of the specific molecule GSDMD in parallel with neuronal pyroptosis. Under treatment with perampanel, the immunofluorescence intensity of GADMD in neurons decreased compared with that in the SAH group, which indicates alleviation of neuronal pyroptosis at 24 h post-SAH.

### 3-TYP partially reverses the alleviating effect of perampanel on neurological deficits and brain edema after SAH

**Figure 4:**
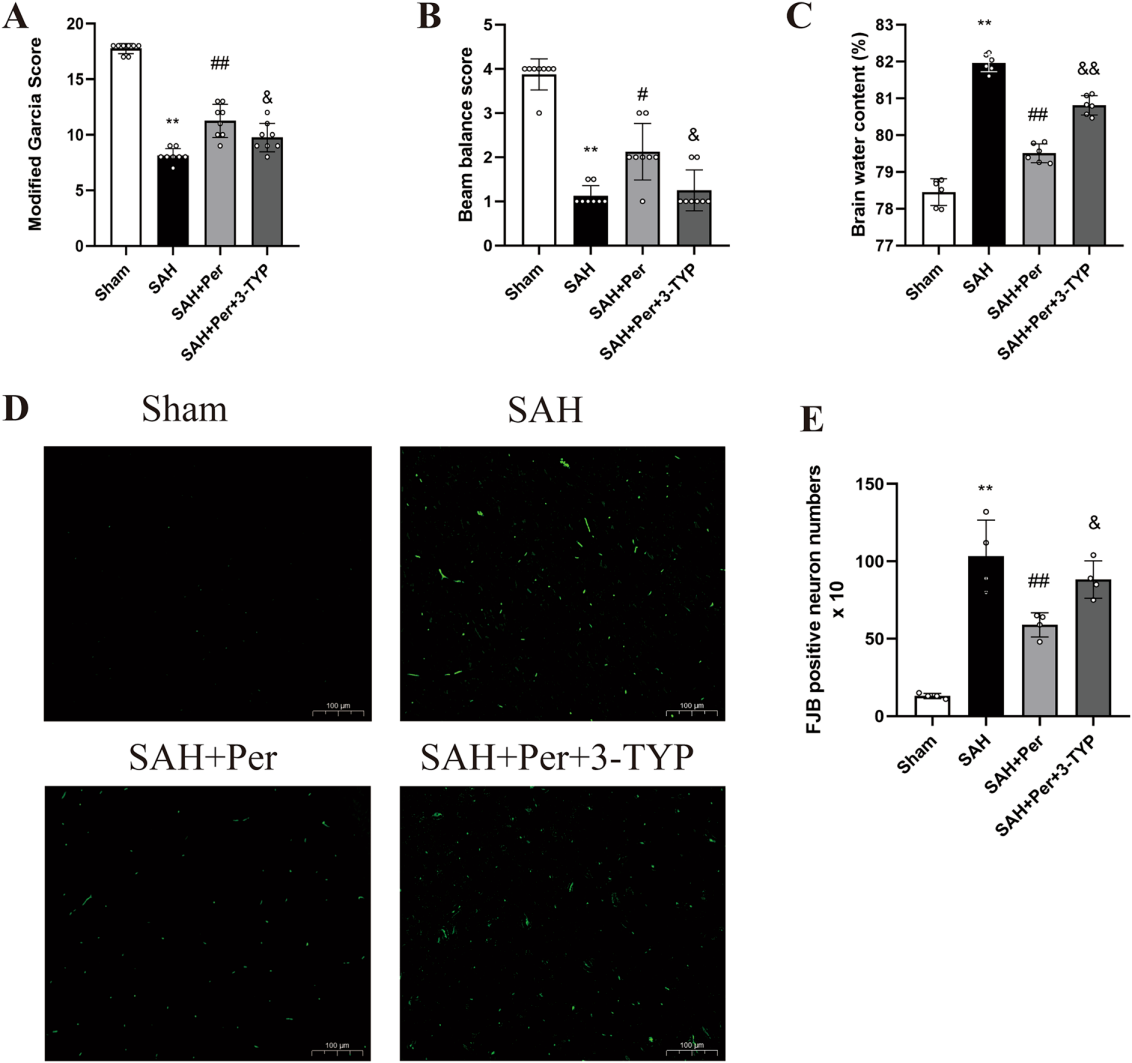
Perampanel improves the neuroscores and reduces brain water content and the degeneration of neurons, and these effects were reversed by 3-TYP. (**A–C**) Quantitative analysis of Modified Garcia Score (n = 8), Beam balance score (n = 8) and Brain water content (n = 6). (**D**) Representative images of FJB straining in the right temporal cerebral cortex of rats (magnification: 10X; scale bar: 100 µm). (**E**) Quantitative measurement of FJB staining (n = 4).

According to the modified Garcia scoring and beam balance test (Fig. 4A,B), treatment with 3-TYP aggravated the neurological deficit compared to the perampanel treatment group. Moreover, FJB staining (Fig. 4D,E) indicated that the positive FJB cell count of the SAH + Per + 3-TYP group increased compared to that of the SAH + Per group, which indicates that 3-TYP aggravates the generation of neurons in the ipsilateral cerebral cortex. Additionally, the BWC results (Fig. 4C) show that 3-TYP partially reverses the alleviating effect of perampanel on brain edema, which represents the degree of damage to the blood‒brain barrier. The above results suggest that 3-TYP partially reverses the alleviating effect of perampanel on neurological deficits and brain edema after SAH.

### 3-TYP partially reverses the alleviating effect of perampanel on oxidative stress and neuronal pyroptosis after SAH

**Figure 5:**
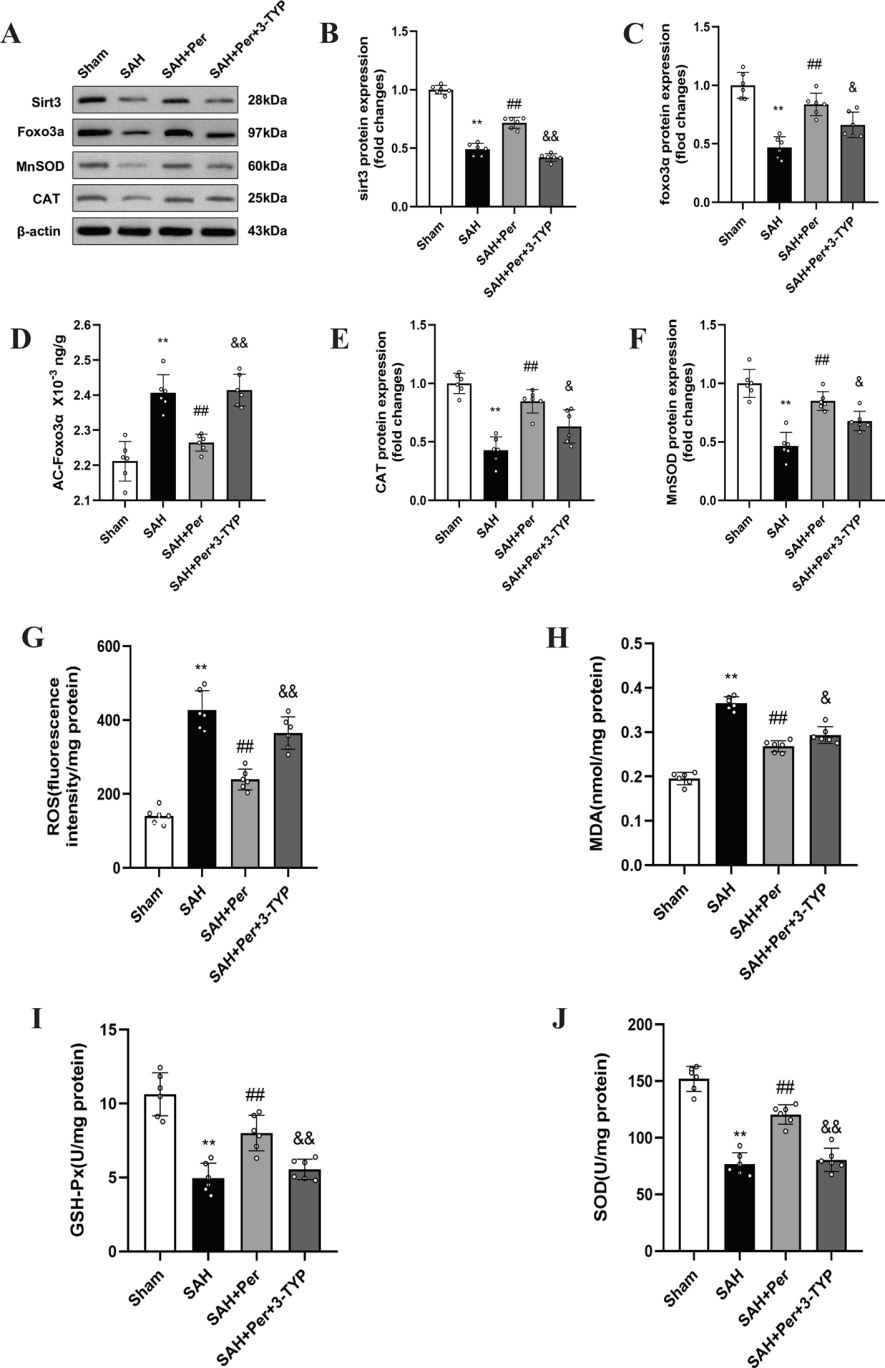
The effect of perampanel on reducing oxidative stress at 24 h after SAH was reversed by 3-TYP, and 3-TYP also partially reversed the effect of perampanel on the sirt3-foxo3a pathway. (**A**) Representative western blot images of SIRT3, FOXO3a, CAT, and MnSOD. (**B–F**) Quantitative analysis of the protein expression levels of SIRT3, FOXO3a, AC-FOXO3a, CAT, and MnSOD in each group (n = 6). (**G**) Quantitative analysis of the ROS assay (n = 6). (**H–J**) Quantitative analysis of MDA, SOD, and GSH-Px detected by ELISA (n = 6). Bars represent the mean ± SD; *P < 0.05, **P < 0.01 vs Sham group; ^#^P < 0.05, ^##^P < 0.01 vs. SAH group; ^&^P < 0.05, ^&&^P < 0.01 vs. SAH + Per group.

**Figure 6:**
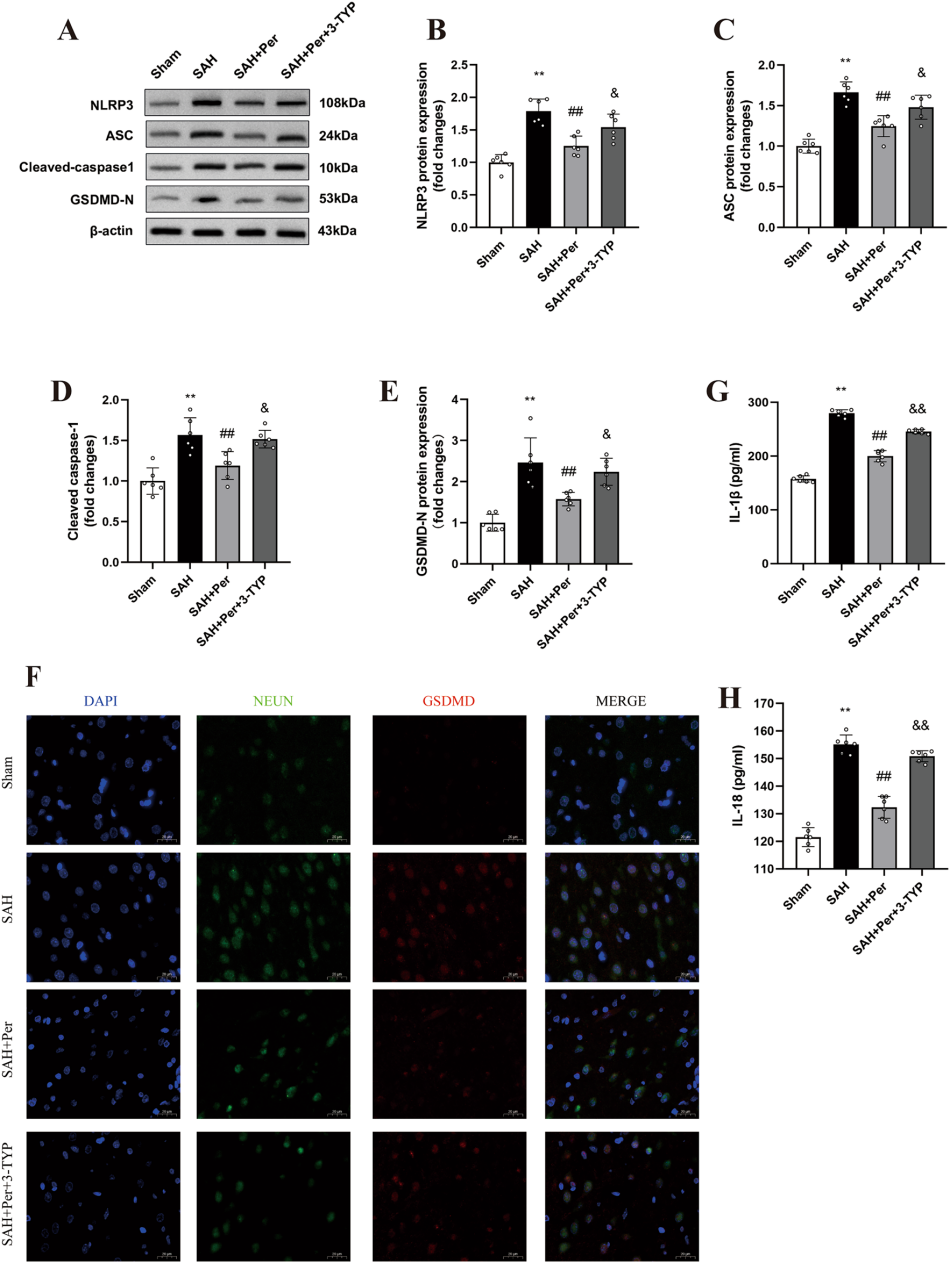
Perampanel alleviates NLRP3 inflammasome-induced neuronal pyroptosis and neuroinflammation at 24 h after SAH, and these effects were reversed by 3-TYP. (**A**) Representative western blot images of NLRP3, ASC, cleaved caspase-1, and GSDMD-N. (**B–E**) Quantitative analysis of the protein expression levels of NLRP3, ASC, cleaved caspase-1, and GSDMD-N in each group (n = 6). (**F**) Double immunofluorescence staining revealed variations in GSDMD (red) in neurons (NeuN, green) in different groups (n = 4). (Magnification: 40X; Scale bar: 20 µm). (**G, H**) Quantitative analysis of the levels of the inflammatory factors IL-1β and IL-18 was detected by ELISA (n = 6).

Compared to those in the SAH + Per group, the content of ROS and MDA in the SAH + Per + 3-TYP group showed significant increases by the ROS assay (Fig. 5G) and ELISA results (Fig. 5H), while the activity of SOD and GSH-Px showed decreases (Fig. 5I,J). The WB results showed (Fig. 5E,F) that the expression of Mn-SOD and CAT in the SAH + Per + 3-TYP group decreased when compared to that in the SAH + Per group. These results indicate that 3-TYP partially reversed the alleviating effect of perampanel on oxidative stress after SAH. WB (Fig. 6B–E) revealed a significant increase in the expression of NLRP3, ASC, cleaved caspase-1, and N-GSDMD in the SAH + Per + 3-TYP group compared with the SAH + Per group. The ELISA results (Fig. 6G,H) showed increases in the contents of IL-1β and IL-18 in the SAH + Per + 3-TYP group compared with those in the SAH + Per group, which indicates the aggregation of pyroptosis and neuroinflammation in the ipsilateral cerebral cortex after SAH. In line with the WB and ELISA results, IF double staining (Fig. 6F) showed that the immunofluorescence intensity of GSDMD in the neurons of the SAH + Per + 3-TYP group was higher than that in the neurons of the SAH + Per group, which indicates the aggravation of neuronal pyroptosis after SAH. These findings indicate that 3-TYP partially reversed the alleviating effect of perampanel on neuronal pyroptosis after SAH.

## Discussion

**Figure 7:**
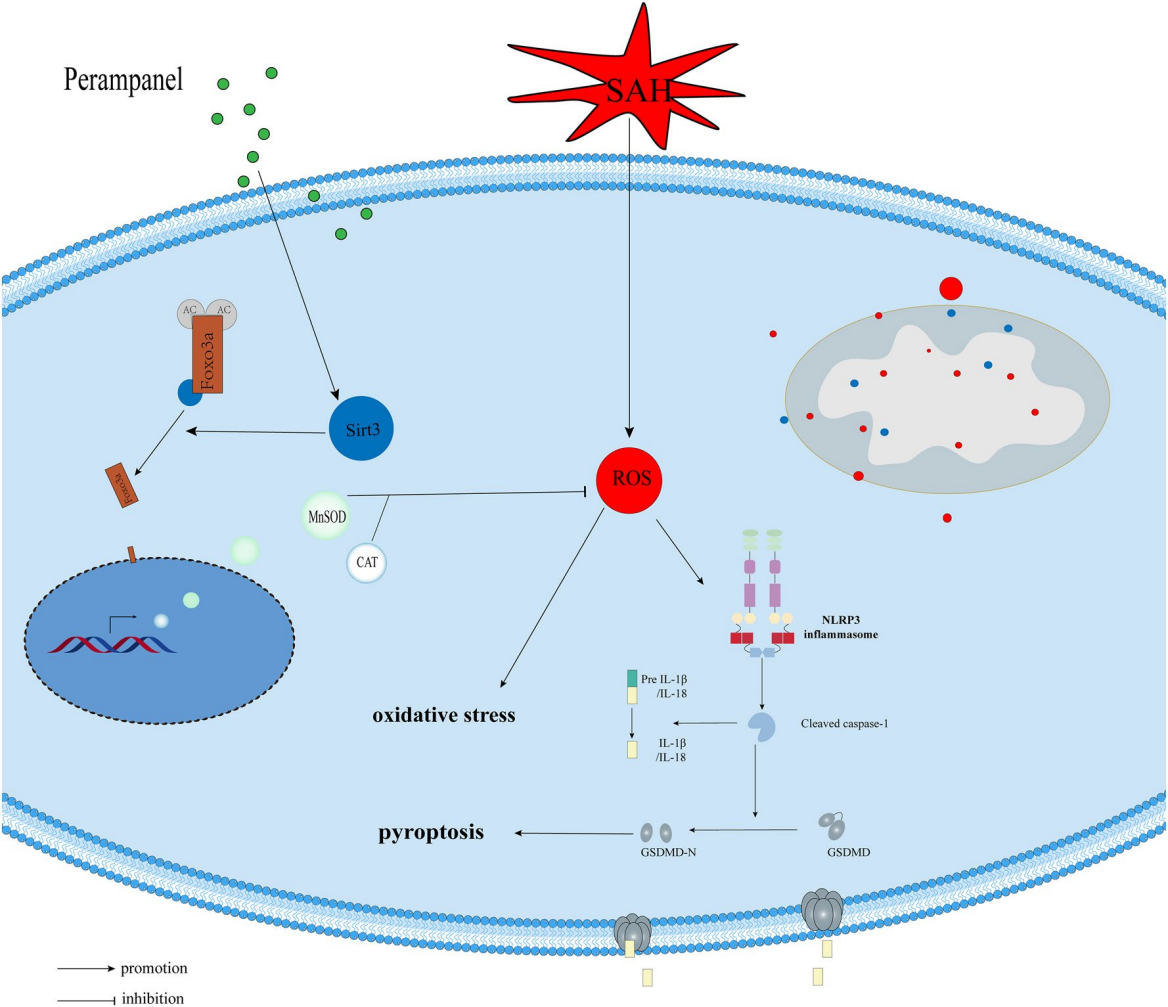
A schematic illustration of the mechanisms by which perampanel reduces early brain injury after subarachnoid hemorrhage. At 24 h after subarachnoid hemorrhage, Sirt3 and FOXO3A expression decreased, and deacetylated FOXO3a was reduced, along with redox dynamic equilibrium being broken; ROS were dramatically increased, which activated the NLRP3 inflammasome, aggravating pyroptosis and neuroinflammation. However, perampanel attenuates pyroptosis and neuroinflammation by activating the SIRT3-FOXO3a-mediated antioxidant damage pathway and accelerating ROS clearance.

We investigated the neuroprotective effects of perampanel in SAH and explored the underlying mechanism of oxidative stress and neuronal pyroptosis during SAH-post EBI in this study (Fig. 7). The following are the major novel findings: (1) the SIRT3-FOXO3α axis in the ipsilateral brain of rats was significantly inhibited 24 h post SAH; (2) oral perampanel treatment significantly reduced oxidative stress, as well as neuronal pyroptosis, in SAH-induced EBI, with the improvement of neurological function; (3) the therapeutic effect of perampanel was strongly associated with upregulation of the level of SIRT3 and deacetylated FOXO3α in the 24 h-post-SAH rat brain; and (4) blockade of SIRT3 partially offset the beneficial effects of perampanel on neurological functions, oxidative stress and pyroptosis. Collectively, our data suggest that perampanel exerts neuroprotective effects by alleviating oxidative stress and pyroptosis in EBI after SAH, which is probably due to the involvement of the SIRT3-FOXO3α signalling pathway.

Oxidative stress is the major pathological feature in the early stage after SAH, and involved in the development of early brain injury, neuronal apoptosis and pyroptosis, and cerebral vasospasm^23,24^. There is an imbalance in the brain that favors the generation of reactive oxygen species (ROS) rather than being neutralized by the intrinsic antioxidant system in experimental animal models and after human SAH^23^. Anti-oxidative stress is an effective way to improve the prognosis of the SAH model. According to a previous study, the levels of ROS and peroxide MDA, 8-OHdG, and PCO were increased, while the activities of the antioxidant enzymes GSH-Px and SOD were decreased after subarachnoid haemorrhage^25^. Han et al.found that an extract from plants significantly reduced MAD levels, increased the SOD, catalase (CAT), and GSH-Px levels after SAH, and reduced many EBI-related indicators (brain edema, BBB disruption, and neurological score)^26^. In our study, perampanel reduced the redox imbalance after SAH, decreased the level of lipid peroxides MDA, and increased the levels of antioxidant enzymes including MnSOD2 and CAT, thus accelerating the clearance of ROS and alleviating brain edema and neurological impairment. When the SIRT3 inhibitor 3-TYP was administered, the antioxidative stress of perampanel was reversed, as shown by the increase in MDA and decrease in MnSOD2 and CAT, along with the aggravation of brain edema, neuronal degeneration, and neurological impairment.

Based on previous definition as caspase-1-dependent proinflammatory programmed cell death, pyroptosis is now characterized as the formation of pyroptotic pores mediated by gasdermin family proteins. These pyroptotic pores cause cell swelling and rupture, and thus the outflow of cell contents and related inflammatory factors, initiating and promoting the inflammatory response^27,28^. According to growing scientific evidence, pyroptosis is one of the main pathological processes in neurologic deficiency caused by SAH-post EBI^20,29,30^. Neuronal pyroptosis has been shown in the brain cortex and hippocampus after SAH and is associated with oxidative stress injury^5,30^. The NLRP3 inflammasome, a multiprotein complex, can cleave caspase-1 to activate it. Activated caspase-1 not only induces pyroptotic pore formation mediated by GSDMD-N, but also promotes the maturation and release of IL-1β and L-18, which aggravate neuronal inflammation and lead to irreversible neuronal death. In our study, the expression of GSDMD-N, indicating the degree of pyroptosis, significantly accumulated at 24 h post SAH, which is compatible with a previous study^30^. However, oral perampanel ameliorated pyroptosis during SAH-post EBI, which was reversed by the SIRT3 inhibitor 3-TYP. Currently, inflammasome activation is considered to be the crucial link in the occurrence of pyroptosis. A variety of factors can affect the activation of the NLRP3 inflammasome, of which the most important factor is ROS^7,30^. The generation of ROS is a characteristic of oxidative stress, and increasing evidence has shown that ROS overload is the primary mediator that causes activation of the NLRP3 inflammasome following SAH^30^. Our study also showed that a reduction in ROS content was accompanied by a reduction in NLRP3 inflammasome activation, along with a reduction in neuronal pyroptosis and neuroinflammation after SAH. Current studies have shown that a variety of inflammasomes are involved in the process of neuronal cell pyroptosis, such as AIM2, NLRP1, and NLRP3, after subarachnoid hemorrhage, among which NLRP3 is the best studied. Notably, whether other inflammasomes, such as NLRP1 and AIM2, are involved in the alleviating effect of perampanel on neuronal pyroptosis remains to be further investigated.

A class of nicotinamide adenine nucleotide-dependent protein deacetylases known as sirtuins (SIRTs), among which SIRT3 primarily exists in mitochondria and has a variety of functions as a stress-related deacetylase. In brain injury research, SIRT3 is associated with mitochondrial homeostasis, oxidative stress, inflammation, and autophagy^7^. According to a previous study, SIRT3 decreased with increasing ROS in SAH-induced EBI^26^. SIRT3 mRNA and protein expression decreased significantly at 8 h after SHA and decreased to the lowest level at 24 hours^31^. According to previous studies^32,33^, EBI was much worse at 24 h after experimental SAH than at other time points, damaged by neuronal apoptosis, brain edema, inflammation, etc. Therefore, we chose 24 h post modelling to investigate the effect of SIRT3 on early brain injury after SAH. Consistent with a previous study, our research showed that the NLRP3 inflammasome was significantly activated at 24 h post-SAH^30^. Accompanied by a sharp increase in the level of NLRP3, we found that the expression of SIRT3 markedly decreased, corresponding to the severity of EBI. In a model of diabetic intracerebral hemorrhage, SIRT3 activation can decrease the levels of NLRP3 and IL-1β by deacetylating MnSOD and clearing ROS, thus reducing neuronal damage^34^. In the depression model, SIRT3 can reduce ROS and activate NF-kB signals in the hippocampus of mice, thereby alleviating NLRP3-induced pyroptosis^35^. Meanwhile, our study demonstrated that perampanel restored SIRT3 expression and promoted ROS clearance, alleviating NLRP3-induced pyroptosis and inflammation and thus alleviating brain edema and neurological dysfunction in rats with SAH.

FOXO3α, as a downstream target of SIRT3, plays an essential role in antioxidative stress. When the cell suffers from oxidative stress, FOXO3α deacetylation increases and decreases its phosphorylation and ubiquitin to maintain its structural and functional stability^36^. Previous studies have shown that the sites at which FOXO3α is acetylated by SIRT3 are located at lysine 271 and lysine 290^37^. Activated (deacetylated) FOXO3α can regulate the expression of the antioxidant enzymes, MnSOD, CAT, and PGC-1a, at the transcriptional level; the former two regulate intracellular redox levels, and PGC-1a can regulate mitochondrial respiration and biogenesis, thus reducing ROS. In microglia, overexpression of SIRT3 increased FOXO3α expression and nuclear translocation, which alleviated the oxidative stress damage of microglia by promoting the expression of the antioxidants MnSOD and CAT; in contrast, knockdown of the SIRT3 gene reduced FOXO3α expression and activation^19^. We demonstrated that SIRT3 and FOXO3α expression was reduced while ROS increased after SAH. Meanwhile, increased acetylated FOXO3a (AC-FOXO3α) indicates a decrease in SIRT3 activity after SAH. When perampanel was administered, the decrease in SIRT3 expression and activity was alleviated, as well as the indexes of oxidative stress and pyroptosis, while the therapeutic effect of perampanel was reversed after the administration of the SIRT3 inhibitor 3-TYP. These results show that perampanel alleviated oxidative stress and pyroptosis, at least, partly through the SIRT3-FOXO3α pathway after SAH. In addition, a study proved that a phenolic compound mainly in cereals attenuates hydrogen peroxide-induced oxidative stress and apoptosis through the SIRT3-FOXO3α signalling pathway^38^. Similarly, another study found that the SIRT3/FOXO3α pathway plays a neuroprotective role, which is associated with antioxidant stress and saving mitochondrial function^39^. Previous studies have shown that SIRT3 deacetylates FOXO3α directly by physical binding, and deacetylated FOXO3α increases the expression of the antioxidant gene MnSOD to reduce diabetes-induced myocardial injury^40^. A compound extracted from sesame seeds reduces oxidative damage to nerve cells through the SIRT1/SIRT3/FOXO3 pathway^41^. The results show that FOXO3α upregulates endogenous antioxidant enzymes, including MnSOD and CAT, and participates in ROS detoxification, which is concordant with our results. A previous study has also shown that the SIRT6-FOXO3α pathway alleviates SAH-induced neuroinflammation^42^, which further indicates that FOXO3α plays an essential role in anti-inflammatory and antioxidative stress after SAH. Importantly, our study demonstrated for the first time in vivo that the SIRT3-FOXO3α pathway plays a neuroprotective role after subarachnoid hemorrhage, which is related to antioxidant stress and neuronal pyroptosis.

**Figure 8:**
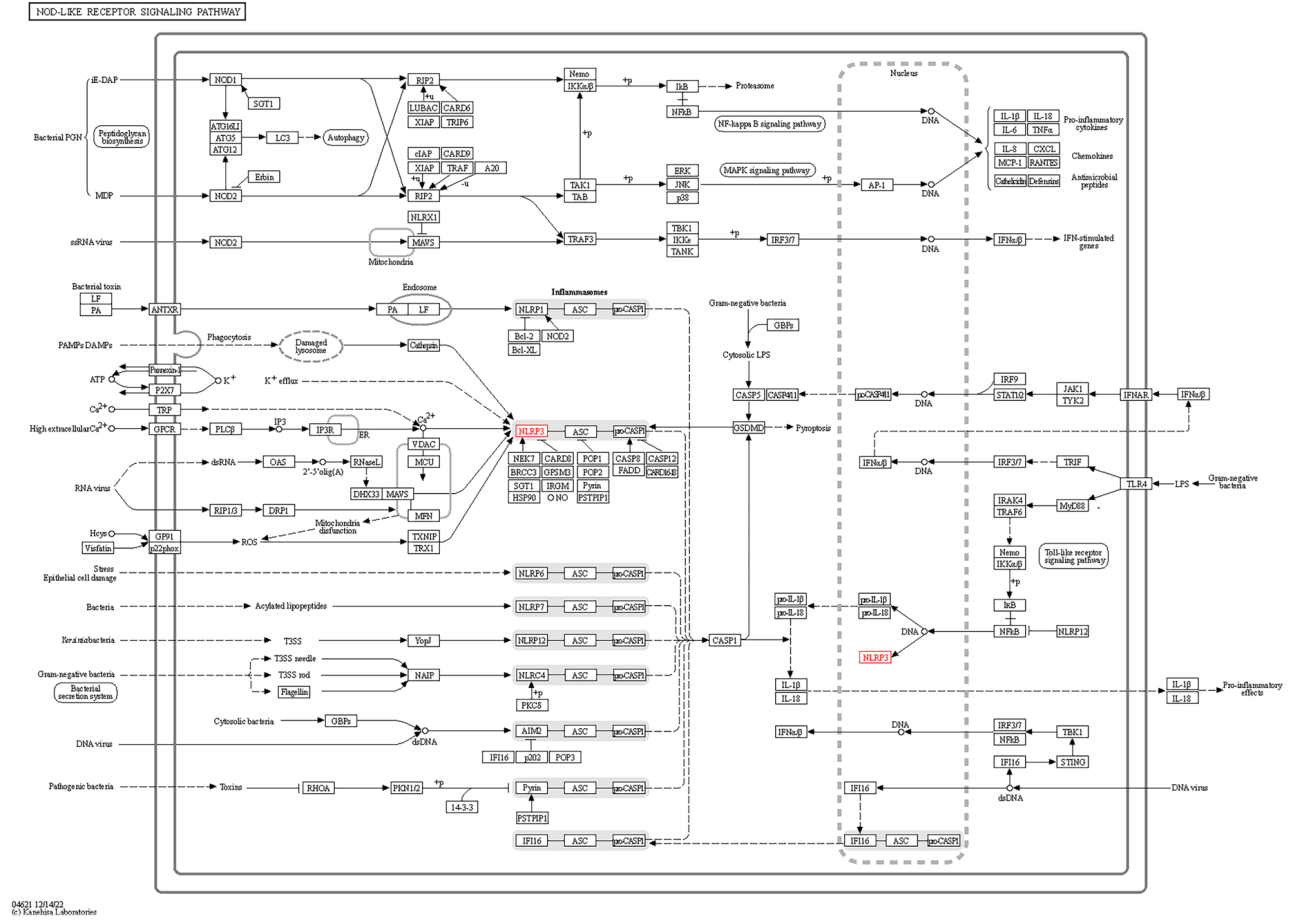
Signaling pathway of NLRP3 inflammasome-mediated pyroptosis from the Kyoto Encyclopedia of Genes and Genomes (KEGG) (https://www.kegg.jp).

SIRT3 and FOXO3α are closely associated with mitochondrial respiration, which promotes the maintenance of mitochondrial homeostasis^39,43^. During oxidative stress, mitochondria are the main source of ROS, and the breakage of the mitochondrial respiratory chain leads to excessive production of ROS^25^. In this study, it remains to be further investigated whether the reduction in the production of ROS caused by perampanel maintains mitochondrial homeostasis through the SIRT3 pathway. In addition, the role of perampanel in the late prognosis of SAH needs to be further investigated. Notably, as suggested by the KEGG signaling pathway map (shown in Fig. 8), many factors can influence NLRP3 inflammasome expression and activation, and we cannot determine whether perampanel reduced activated NLRP3 through pathways other than ROS. It is a reasonable assumption that perampanel can antagonize the AMPA receptor to affect intracellular calcium levels, thus affecting the expression and activation of NLRP3. To fully elucidate the mechanism of perampanel on neuronal pyroptosis after SAH, more investigations are warranted. In this experiment, we administered the drug (perampanel) 2 h in advance so that the rats had enough time to restore calm after intragastric administration to reduce the effects of preoperative stress and to avoid further damage caused by intragastric administration after the operation. Additionally, there are still the following shortcomings in this study. First, whether perampanel reduces neuronal pyroptosis in other ways remains to be further discussed. Second, we focused on the anti-pyroptosis and antioxidative stress effects of perampanel after SAH, but we cannot exclude other functions of perampanel, such as the regulation of ferroptosis and necrosis.

## Conclusion

In conclusion, our study has shown that oral perampanel can reduce oxidative stress and neuronal pyroptosis in EBI after SAH through the SIRT3-FOXO3α pathway. This study highlights the application value of perampanel in subarachnoid hemorrhage and lays a foundation for clinical research and later transformation of perampanel in SAH.

### Supplementary Information

Supplementary Information.

## Data Availability

All data generated or analyzed during this study are included in this article. The datasets used and/or analyzed during the current study are available from the corresponding author on reasonable request.
