# Supplementary material for: Perampanel attenuates oxidative stress and pyroptosis following subarachnoid hemorrhage via the SIRT3/FOXO3α pathway
Source: Sci Rep. 2023 Dec 3;13:21320. doi: 10.1038/s41598-023-48802-1 (PMC10694148; doi:10.1038/s41598-023-48802-1)

Fig.3WB

Sirt3


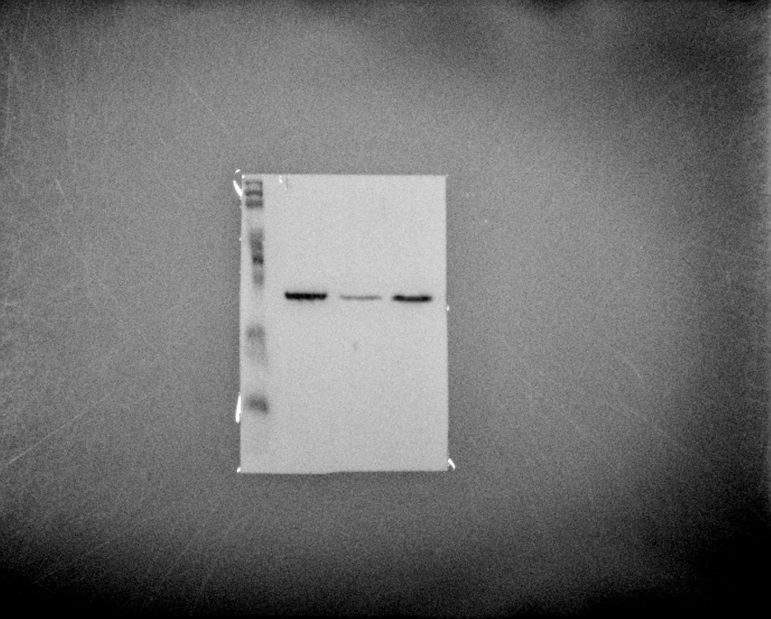


Foxo3α


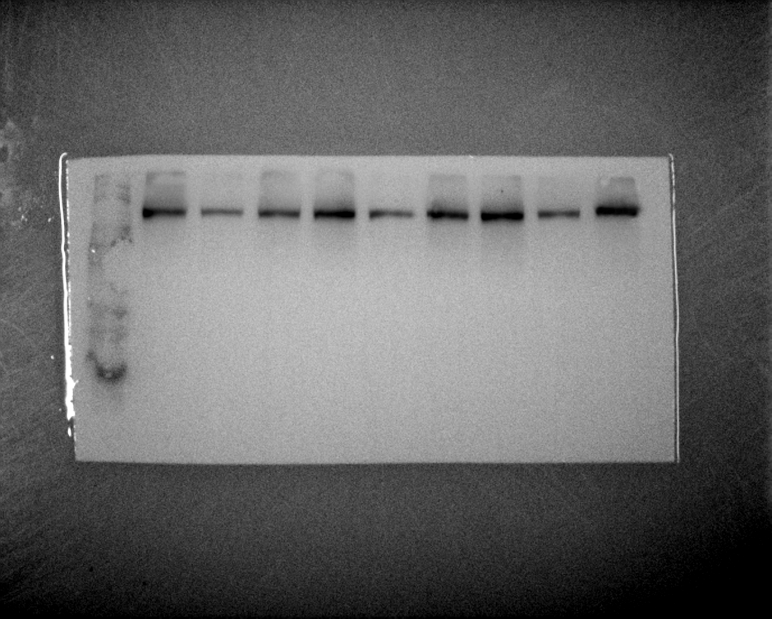


β-actin


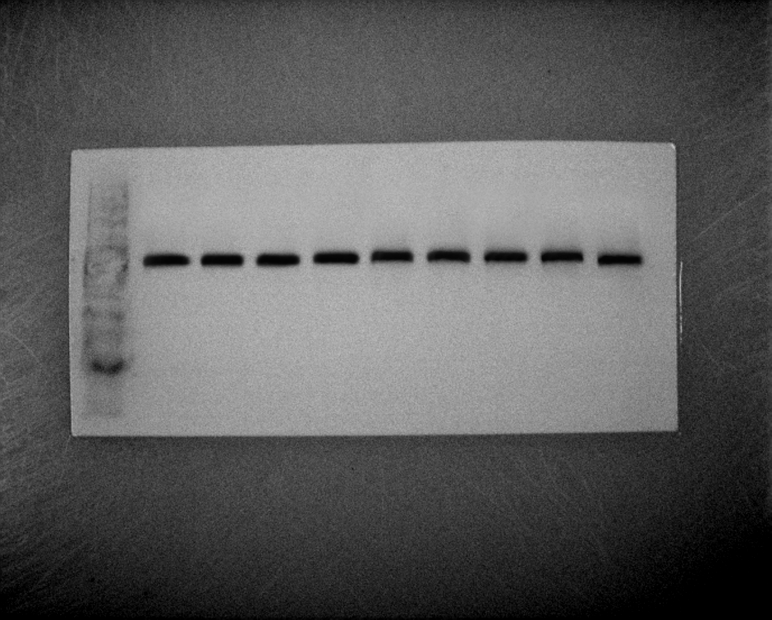


Fig.5WB

Sirt3


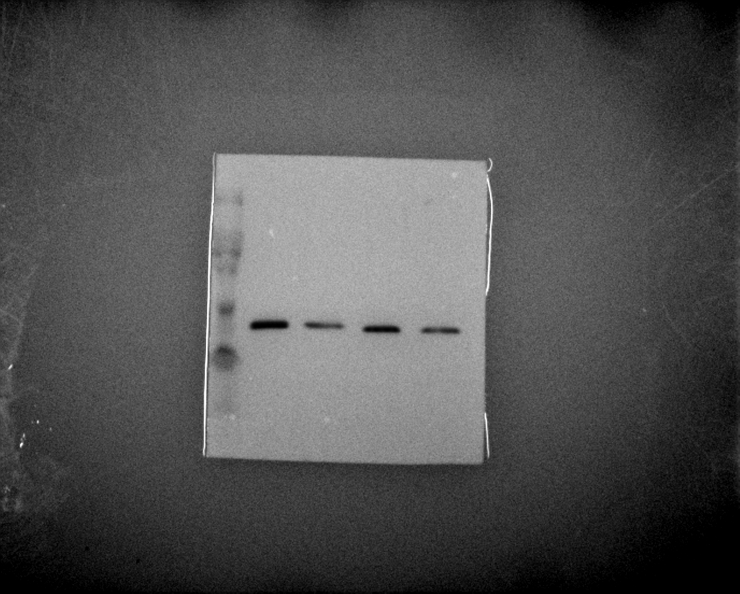


Foxo3α


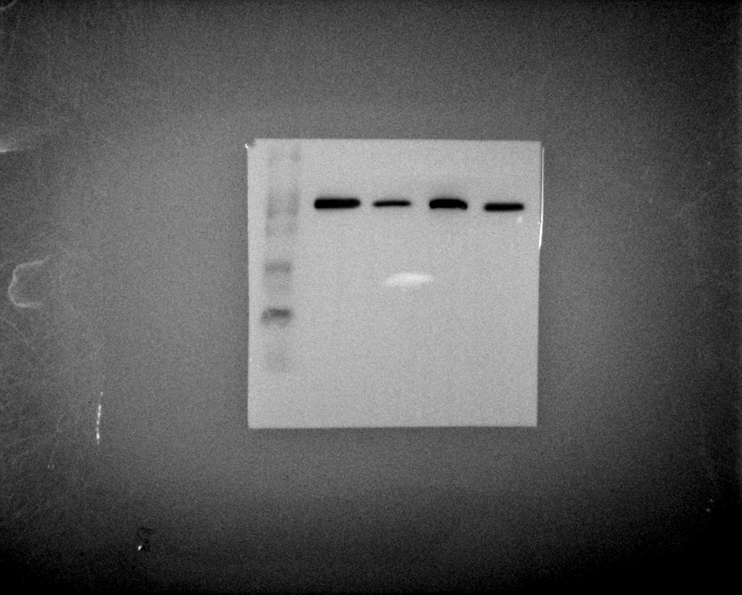


MnSOD


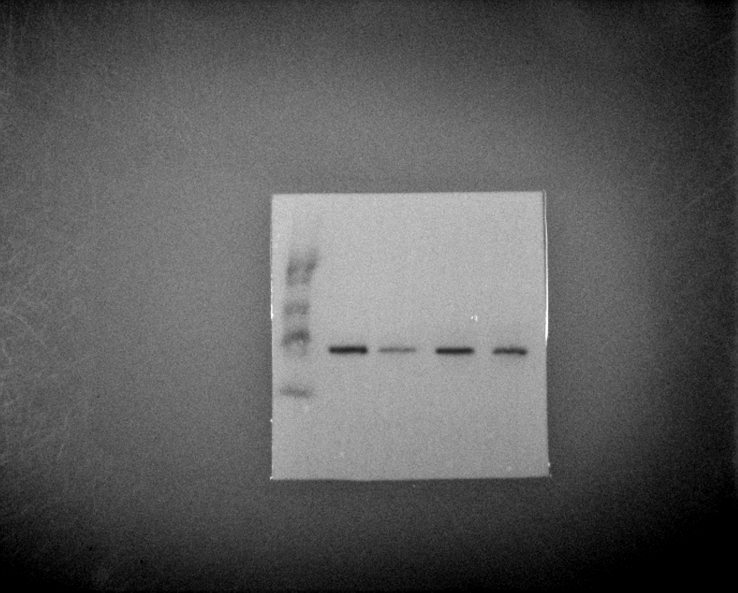


CAT


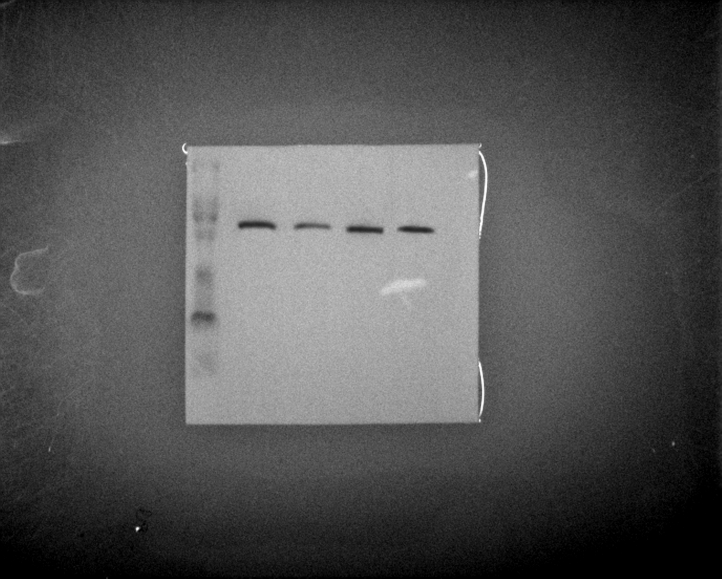


β-actin


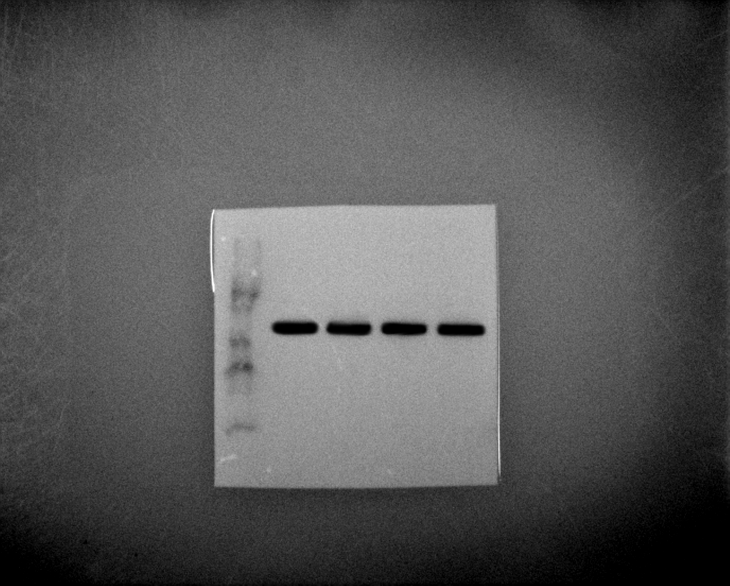


Fig.6WB

NLRP3


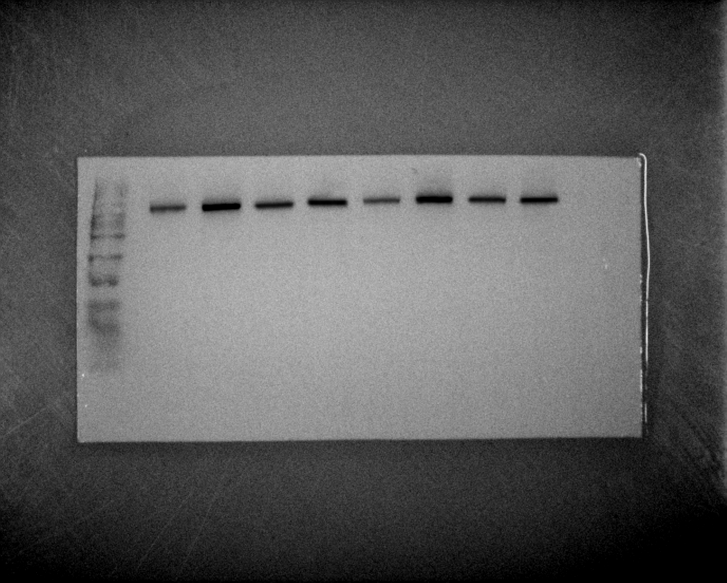


ASC


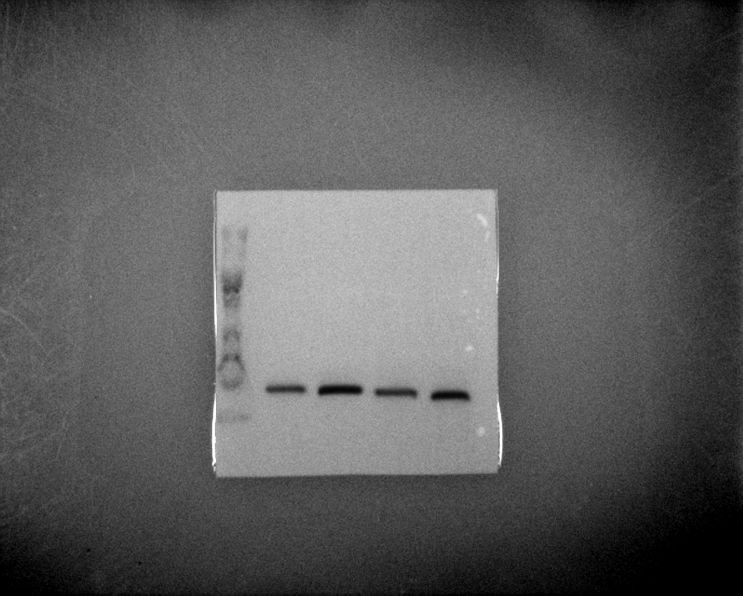


Cleaved-caspase1


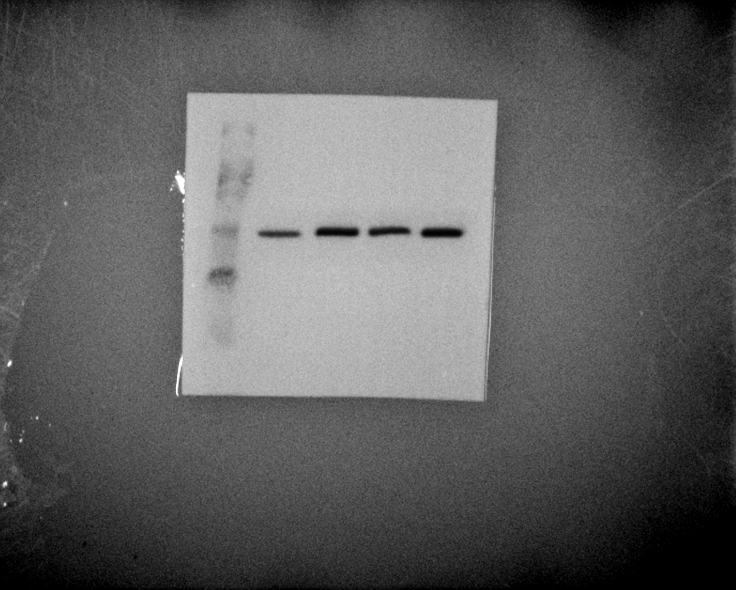


GSDMD-N


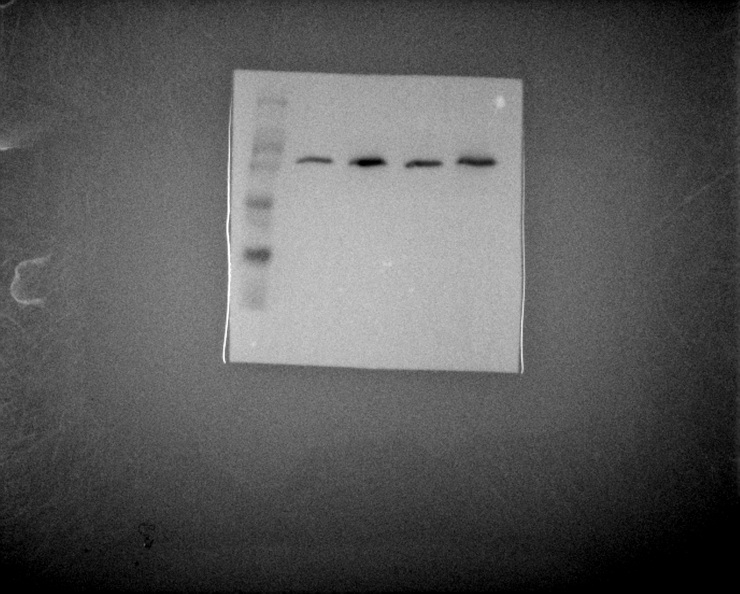


β-actin


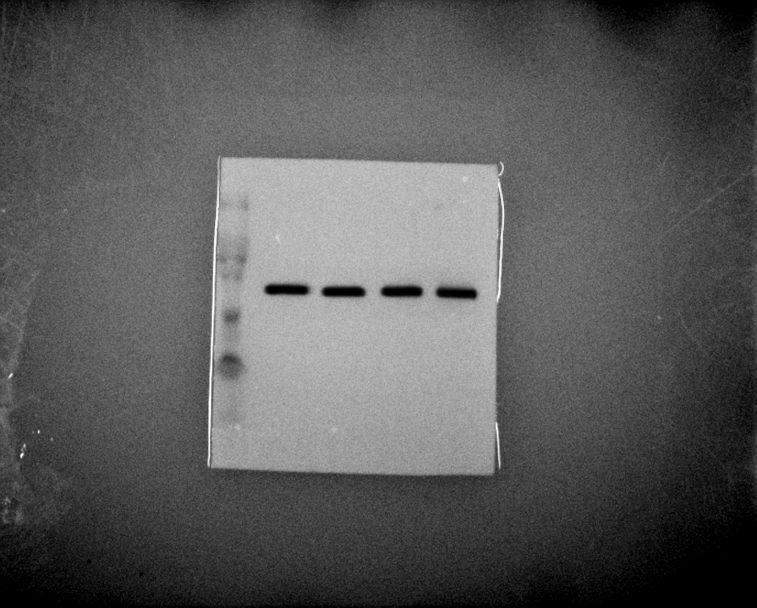

Supplement: Supplementary file 1 — Supplementary Information. [file 41598_2023_48802_MOESM1_ESM.docx]
